# A transgenic Drosophila melanogaster model to study HTLV-I oncoprotein Tax-driven leukemogenesis in vivo

**DOI:** 10.1186/1742-4690-12-S1-O40

**Published:** 2015-08-28

**Authors:** Margret Shirinian, Zakaria Kambris, Lama Hamadeh, Chloé Journo, Caroline Grabbe, Renaud Mahieux, Ali Bazarbachi

**Affiliations:** 1 Department of Internal Medicine, Faculty of Medicine, American University of Beirut, Beirut, Lebanon; 2 Department of Biology, American University of Beirut, Beirut, Lebanon; 3 Equipe Oncogenèse Rétrovirale, Lyon, Cedex 07, France; 4 Equipe labellisée “Ligue Nationale Contre le Cancer”, Lyon, Cedex 07, France; 5 Centre international de recherche en infectiologie, INSERM U1111 -CNRS UMR5308, Lyon, Cedex 07, France; 6 INSERM U1111 Ecole Normale Supérieure de Lyon, 46 allée d'Italie, 69364 Lyon, Cedex 07, France; 7 Université Lyon 1, LabEx ECOFECT -Eco-evolutionary dynamics of infectious diseases, 69364 Lyon, Cedex 07, France; 8 Department of Molecular Biology, Umea University, Umea, Sweden; 9 Department of Anatomy, Cell Biology and Physiological Sciences, American University of Beirut, Beirut, Lebanon

## 

Adult T-cell Leukemia/Lymphoma is an aggressive malignancy caused by HTLV-1 infection. HTLV-2 is genetically related to HTLV-1, but does not cause a malignant disease. The HTLV-1 Tax (Tax-1) viral transactivator is required for HTLV-1 expression and modulates the classical and non-canonical NF-κB pathways. Interaction of Tax-1 with IKKγ/NEMO results in constitutive activation of NF-κB in HTLV-1 infected cells, and contributes to HTLV-1-driven leukemogenesis. Tax-1 transgenic mice develop leukemia, lymphomas or spontaneous osteolytic bone metastases demonstrating Tax-1 oncogenic properties in vivo. However, the cellular pathways and the partners involved in vivo have not been described. HTLV-2 Tax (Tax-2) has properties different from Tax-1, including different post-translational modifications and different intracellular localization. Thanks to the availability of collection of mutants and RNAi lines, Drosophila melanogaster allows simple and exhaustive genetic screens. We generated transgenic Drosophila models expressing either Tax-1 or Tax-2 in the compound eye and plasmatocytes (leukocyte-like cell). We demonstrate that Tax-1 but not Tax-2 induces a perturbation of the crystalline array of the ommatidia and increase in plasmatocyte proliferation indicating that Tax-1 but not Tax-2 has transforming potential in Drosophila. We further show that induction of the eye phenotype is primarily dependent on Kenny, the Drosophila homolog of IKKγ/NEMO, upstream of Relish (NF-κB) activation. Using this model we were able to identify a novel post-translational modification which Tax-1 undergoes in addition to the well-known ubiquitylation and SUMOylation. This novel Tax post-translational modification was confirmed in HTLV-I transformed cell lines. Altogether, these results show that the Drosophila system is useful for dissecting the molecular mechanisms of HTLV-1-induced cell transformation in vivo.

